# Multiscale Resource Selection for a Reintroduced Elk Population

**DOI:** 10.3390/ani16071076

**Published:** 2026-04-01

**Authors:** Braiden A. Quinlan, Brett R. Jesmer, Jacalyn P. Rosenberger, William Mark Ford, Michael J. Cherry

**Affiliations:** 1 Department of Fish and Wildlife Conservation, College of Natural Resources and Environment, Virginia Tech, Blacksburg, VA 24060, USA; brettjesmer@vt.edu; 2 Virginia Department of Wildlife Resources, Marion, VA 24354, USA; jackie.rosenberger@dwr.virginia.gov; 3 U.S. Geological Survey, Virginia Cooperative Fish and Wildlife Research Unit, Blacksburg, VA 24061, USA; wmford@vt.edu; 4 Caesar Kleberg Wildlife Research Institute, Texas A&M University-Kingsville, 700 University Blvd., MSC 218, Kingsville, TX 78363, USA; michael.cherry@tamuk.edu

**Keywords:** *Cervus canadensis*, continuous-time movement modeling, habitat selection, reintroduction, restoration, surface coal mine, translocation

## Abstract

Patterns of resource selection are driven by the decision-making processes of animals occurring at multiple scales from where to establish a home range (i.e., second order selection) to which resource patches to use within the home range (i.e., third order selection). Elk (*Cervus canadensis*) were reintroduced to southwestern Virginia, USA, from 2012 to 2014 following successful translocations onto reclaimed surface coal mines in the region. We sought to understand how elk have acclimated following their translocation using location data from GPS-collared adult female elk (*n* = 33) collected from 2019 to 2022 along with remotely sensed terrain and land cover data. We utilized continuous-time movement models paired with generalized linear mixed-effects modeling to describe seasonal resource selection at second and third orders. At both scales of selection and throughout the year, female elk selected reclaimed surface mines, conifer forests, ridgetops, and areas with lower terrain roughness, while avoiding mixed hardwood and oak (*Quercus* spp.) forests. Unmined open land was only selected at the third order during periods of forage scarcity (i.e., winter) and increased metabolic requirements (i.e., late gestation). Although surface coal mining leaves legacy environmental impacts on the landscape, management of these sites provides benefits to elk and maintains open habitat that is otherwise limited.

## Simple Summary

Animals must make decisions about where to sleep, feed, and hide, amongst others, and these decisions are based on how the animals perceive their environment throughout the year. We used location data from GPS-collared female elk reintroduced to southwestern Virginia to determine what resources they select seasonally. We investigated two levels of selection: second order, the establishment of a home range, and third order, resource patches to use within the home range. We found female elk selected reclaimed surface coal mines, conifer forests, ridgetops, and areas with lower terrain roughness, while avoiding mixed hardwood and oak forests at both orders. Unmined open land was only selected at the third order during periods of forage scarcity (i.e., winter) and increased metabolic requirements (i.e., late gestation). During these times, selection for agricultural areas, such as hay fields, cattle pasture, and croplands could result in human-wildlife conflict. In the forest-dominated central Appalachian Mountains, reclaimed mines create a mosaic of structurally diverse, grassy or shrub-dominated areas that provide elk with both forage and cover. The management of reclaimed surface coal mines not only provides benefits to elk but may also maintain open habitat that is otherwise limited and rare.

## 1. Introduction

The reintroduction of extirpated species is a fundamental tool in wildlife conservation and has led to the successful recovery of numerous species [1,2]. Successful wildlife reintroductions require empirical knowledge about the movements of reintroduced species [2] which informs habitat management and potential range expansions amongst others. Identifying suitable reintroduction sites depends on understanding the habitat requirements of the focal species and how the reintroduction landscape may satisfy those requirements [2]. Further, knowledge of resource selection elucidates potential for range expansion beyond the reintroduction site as a result of identifying high-quality habitat for the species of interest and can guide habitat management designed to support the species [3]. However, reintroductions, particularly reintroductions of large mammals, can generate human-wildlife conflict (e.g., crop damage by herbivores or predation on livestock by carnivores) [4,5,6]. Thus, quantifying behavioral responses of reintroduced species to spatiotemporal variability in resource availability (i.e., resource selection) may help mitigate potential conflict and ensuring long-term population persistence.

Resource selection reflects the decisions made by individuals based on their life history and cognitive abilities (experience and memory) which, over time, guide movements, resulting in patterns of space use [7]. Factors influencing decisions include food availability [8], intra- and inter-specific competition [9,10], predation risk or human activities [11,12], cover [13,14,15] and reproductive success [16,17]. In addition to these factors, resource selection is further influenced by the availability of resources at multiple spatial scales, such as where an animal chooses to establish a seasonal or home range on the landscape (i.e., second order resource selection) and their movements within the range (i.e., third order resource selection). Hence, analyses at multiple scales to document these factors often are critical to inform management [18,19,20].

For ungulates—hooved mammals—deciding where to establish a range, or area that supports an organism’s life history strategies (second order selection), is thought to be driven by the availability of forage resources across the landscape [21]. Movement decisions within the established range (third order selection), however, are mediated by intra- and inter-specific competition as well as thermal requirements [15,22] and refugia from predators [12,17]. Further, decisions made by animals can be influenced by ecological tradeoffs associated with seasonal and metabolic demands. For instance, in times of resource limitation, animals may tolerate real or perceived predation risk for access to high forage quality and quantity [17]. As energetic requirements fluctuate throughout the year [23], resource selection may change with these fluctuations to take advantage of seasonal resource availability such as new plant growth in the spring or hard mast (i.e., acorns) in the fall [24,25,26]. As such, an understanding of what landscape features animals prioritize across the annual life cycle can aid managers in their decision making. Continued assessments of resource selection, particularly following reintroductions, can therefore provide information about how species are acclimatizing to their new landscape [27].

In addition to resource availability, it is also important to examine how species have adapted to and interact with the topographic environment. Some ungulate species specialize on specific subsets of elevational range and topographic characteristics based on their natural history [28,29,30]. For example, elk (*Cervus candensis*) select differing slope, terrain roughness, and elevation features across the species’ range [13,16,31]. In western North America, elk often select higher elevations and more rugged terrain to reduce predation risk [11,12,31]. Also, in western North America, elk typically utilize higher elevations during the summer and lower elevations during the winter due to forage availability and thermal conditions [31,32,33]. Female elk may use differing topography as a buffer for birth site concealment and protection [17,34]; the bottom of steep valleys and north-facing ravines for cooler microclimates from cold-air drainage where for thermal refuge during hotter weather [35]; or forested ridgetops may serve as easier means to traverse the landscape or as areas to rest and ruminate [36,37,38]. Therefore, elevation and topography may have greater influence on fine-scale habitat utilization in the Appalachian Mountains compared to larger, range-wide selection.

Different land cover types in the central Appalachian Mountains may have various functional attributes relative to elk. Deciduous forests may provide cover from thermal extremes or human disturbance and forage from spring through fall, but during winter, deciduous forests with no leaf cover provide elk with little thermal cover or forage [24,26,39]. Most reclaimed surface coal mines (hereinafter reclaimed mines) in the region are functionally open grasslands and shrub lands in an extended period of arrested succession [40]. Accordingly, reclaimed mines provide a mosaic of open and early-successional habitat embedded within a forest-dominated system, while also including areas of edge and dense stunted woody and shrubby cover [41,42,43]. In the central Appalachian Mountain “Coalfields” region (hereinafter Coalfields), elk use reclaimed mines throughout the year due to their abundance of grassy and forb forage [24,26] and possibly thermal refuge provided by conifers planted during the reclamation process (primarily white pines, *Pinus strobus*) [15,31,44]. Lastly, non-mined open habitats in the region are primarily improved domestic cattle (*Bos taurus*) pasture and hay fields which may provide elk with quality forage but come at the expense of competition with domestic livestock and the potential for human-elk conflict [10]. For the long-term viability of elk in the central and southern Appalachian Mountains, research on elk resource selection at multiple scales is needed to inform current and future habitat management and areas to prioritize for protection.

Our research objective was to examine seasonal resource selection across different spatial scales and biological periods. By doing so, we aimed to provide southwestern Virginia and eastern North American managers with data contributory for decision making regarding elk restoration. Specifically, we aimed to (i) ascertain resource selection for female elk at the seasonal range scale (second order) and within the seasonal range (third order) and (ii) evaluate resource selection during different biological seasons, such as during gestation or post parturition to better understand habitat requirements throughout the annual cycle. Given the topography and landscape composition in the reintroduction zone, we predicted (1) female elk would select reclaimed coalmine surfaces throughout year because forage availability is highest in this cover type throughout the year, (2) female elk would select for gentler topography in an effort to avoid the energetic demands of traversing more rugged areas, and (3) female elk would select forested habitats during the rut (fall) and during the late gestational period (spring) to take advantage of ephemeral, high-quality forage (i.e., hard mast in fall such as acorns, new woody or herbaceous plant growth in spring [24,25,26]).

## 2. Materials and Methods

No artificial intelligence tools were used in this research.

### 2.1. Study Area

**Figure 1:**
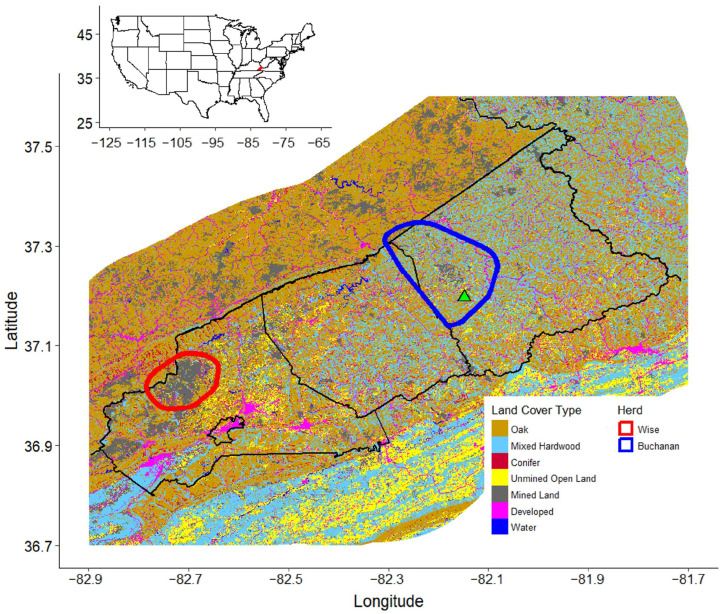
Map depicting the Virginia Elk Management Zone in southwestern Virginia which was comprised of (from left to right) Wise, Dickenson, and Buchanan Counties (black polygons). Reclassified land cover types were oak forests (brown), mixed hardwood forests (light blue), conifer forests (burgundy), unmined open landscapes (yellow), mined landscapes (gray), developed areas (pink), and water (dark blue). Within the Virginia Elk Management Zone were two elk herds, one in northern Wise County (red polygon) and the other in northwestern Buchanan County (blue polygon). The reintroduction site (green triangle) for the 2012, 2013, and 2014 released cohorts was in western Buchanan County.

During 2012–2014, elk (*n* = 75) were translocated from eastern Kentucky, where a large-scale reintroduction had previously occurred, to Buchanan County in southwestern Virginia. The Virginia Department of Wildlife Resources (VDWR) designated Buchanan, Dickenson, and Wise counties as the Virginia Elk Management Zone (VEMZ; [45]; Figure 1). Although elk now occur throughout the VEMZ, there are two main herds: one within and near the original release site in Buchanan County and the other on the Virginia–Kentucky border in Wise County (Figure 1). The VEMZ is in the central Appalachian Mountains’ Plateau physiographic sub-province [46]. This region is dominated by deciduous forests which included oaks (*Quercus* spp.), maples (*Acer* spp.), American beech (*Fagus grandifolia*), yellow-poplar (*Liriodendron tulipifera*), hickories (*Carya* spp.), white ash (*Fraxinus americana*), black cherry (*Prunus serotina*), and basswood (*Tilia americana*) with understory species including flowering dogwood (*Cornus florida*), azaleas/rhododendrons (*Rhododendron* spp.), sassafras (*Sassafras albidum*), mountain laurel (*Kalmia latifolia*), and northern spicebush (*Lindera benzoin*; [47,48]). Although limited in our study area, conifer forests were dominated by natural occurrences of eastern hemlock or eastern white pine that was commonly planted as part of the surface coal mine reclamation process [41,48]. The central Appalachian Mountains have a long history of both deep and surface coal mining, with the latter being more common over the past few decades [49]. Legumes and grasses are common on reclaimed mines including lespedeza (*Lespedeza cuneata*), clovers (*Trifolium* spp. and *Melilotus* spp.), bird’s-foot trefoil (*Lotus corniculatus*), redtop (*Agrostis alba*), tall fescue (*Festuca arundinacea*), rye (*Secale cereale*), cat grass (*Dactylis glomerata*), and several woody plants in addition to eastern white pine, including black locust (*Robinia pseudoacacia*) and autumn olive (*Elaeagnus umbellate*). Although reclaimed mines were the most prominent form of open habitat, other non-mined open habitat in this region was dominated by domestic cattle pastures which generally are vast monocultures of tall fescue. The elevation in our study area ranged from ~180 m to ~1440 m above sea level and topography was characterized as rugged with precipitous slopes and narrow, incised valleys with reclaimed mines and pasture as the major sources of shallow slopes and flat land [43,50,51].

### 2.2. Data Collection and Analytical Methods

We captured adult female elk during winter months (January–March) each year from 2019 through 2022 via darting from vehicles. Further details of capture methods can be found in Quinlan, Rosenberger [39]. Upon capture, we equipped elk with global positioning system (GPS) collars (Advanced Telemetry Systems G5-2D Iridium; Isanti, MN, USA) and ear tags (7.62 cm cattle tags) with a unique number for identification purposes [52].

The movements of adult female elk (*n* = 33) were tracked from January 2019–November 2022. We separated locations into four seasonal distinctions: mid-gestation (1 December–28 February), late gestation (1 March–31 May), calf rearing (1 June–31 August), and breeding (1 September–30 November) for each year. Seasons were based on their biological and energetic significance for female elk while also dividing the year into periods of as similar length as possible. Only individuals with at least 60 locations during a given season were included in our analysis. See Quinlan, Rosenberger [39] for further descriptions of seasonal distinctions and tracking methodologies.

To quantify resource availability (e.g., areas that could have been used by elk), at the second order (where to establish a home range) and third order (which resource patches to use within the home range) [21], we started by separating individuals by their herd within Virginia. Utilizing all locations collected for each individual, we first inspected the variograms of each individual’s movement track to confirm range residency [53,54]. Thereafter, to better emulate elk movement and ranging behaviors, we created lifetime continuous-time movement models individually (using ctmm.guess and ctmm.select) these models reflect movements as a continuous-time stochastic process (ctmm package version 0.6.0 [53] in program R version 4.2.2 [55]). Using the individual-level continuous-time movement models, we generated 99% autocorrelated kernel density estimates (AKDEs) for each elk using the akde function [53]. We established herd ranges (availability area for the second order analyses) using a convex hull around the upper confidence interval of these AKDEs using the gConvexHull function version 0.5-9 [56].

To classify land cover types across our study area, we used two 30 m resolution land cover layers: the National Gap Analysis Project (GAP) Land Cover Data 2011 version 3.0 [57] and the Northeastern Terrestrial Habitats (NTH) layer [58]. We reclassified land cover in ArcMap version 10.8.1 [59] following Ford, McCay [60] and Kniowski and Ford [61] into seven distinct types including: oak forests, cove and mixed mesophytic hardwoods, conifer (primarily natural or planted pine or eastern hemlock), mines (quarries, mines, gravel pits, oil and gas wells, or disturbed and barren lands associated with mining), non-mining open land (livestock pasture, hayfields, cultivated, and other non-mine open lands), developed (communities, roads, and infrastructure), and water (reservoirs, large rivers, or other riparian or wetland area) based on the descriptions of the finer-scale land cover types provided by the datasets. For the study area, we derived the mines land cover type and all adjacent Kentucky land cover from GAP and the remaining habitat types for the rest of the study area from NTH. We combined these land cover raster layers and calculated Euclidean distance to each land cover type in ArcMap [59]. We obtained terrain variables from a digital elevation model (DEM; [62]) and calculated elevation, topographic position index (TPI), terrain roughness (the difference between the maximum and the minimum elevation of a cell and its eight surrounding cells), and slope using the terrain function in the raster package [63] in program R [55]. We used the quadratic form of elevation (elevation^2^) in our models to differentiate moderate elevations from lower and higher elevations.

At both the second and third order, we extracted terrain values and distances to land cover types for each ‘used’ and ‘available’ location for each individual-season-year combination. We scaled and centered extracted values across seasons for each analysis so that the effect sizes of different variables could be compared [64,65]. We checked for collinearity and removed variables that were highly correlated |r| > 0.7 [66]. We used generalized linear mixed-effects models with logistic regression using the function glmer from the lme4 package version 1.1-38 [67] in program R. For each model, the response variable was the location (used or available) with ‘used’ locations assigned a weight of one and ‘available’ locations assigned a weight of 1000 [65,68] and the fixed effects were the land cover and terrain variables. Individual elk were treated as a random intercept to avoid psuedoreplication and obtain less biased estimates.

To build the models for both the second and third order analyses, we created a global model including all land cover types and terrain variables to identify contributory covariates. We began with a null model and continued to add variables from the global model that explained the most variation in the response variable in a forward stepwise fashion. After adding a variable, we checked the variance inflation factors (VIF) to ensure added terms to the model were not correlated (VIF < 4.0; [66]). We continued to add terms to the model from the global model until either all variables were included or adding another term did not result in a lowering of the Akaike information criterion corrected for small sample size (AICc) by four AICc points. We then compared models using Akaike information criterion corrected for small sample size (AICc) and selected the top model that was greater than four delta AICc better than the next model. To evaluate model goodness-of-fit and its predictive power, we used k-fold cross validation (k = 4, 100 repetitions, and 10 bins). If the mean of Spearman’s Correlation Coefficient calculated across all the ‘observed’ folds was greater than 0.8, we considered the model to have good predictive power [69,70].

We employed a sensitivity analysis for both the second and third orders to determine appropriate sample sizes for each analysis. For the second order analysis, we sampled the seasonal range of each by year by season combination by randomly distributing locations (with a weight of one) within the AKDE 99% isopleth. We sampled ‘available’ locations by randomly distributing locations (with a weight of 1000) within the specific herd’s distribution. To determine how many used and available locations were sufficient to characterize use versus availability, we ran the full, or global, model including all variables using a constant 20,000 available locations for each individual but varied the number of used locations spanning from 500 to 2000 at increments of 500. We began by comparing selection (i.e., beta) coefficients and their standard errors between results using 500 and 2000 used locations. If the standard errors overlapped for all variables, 500 locations were considered sufficient. If standard errors did not overlap for at least one variable, we increased the sample size to compare the results from sample size of 1000 to the sample size of 2000. We continued to incrementally compare used location sample sizes until standard errors for all variables overlapped.

After finding the required ‘used’ sample size, we implemented the same methods to determine the available sample size (random available locations). We kept the used sample size constant (determined in the previous step) and compared increasing available sample sizes ranging from 10 to 50 per used in increments of 10. Because we considered GPS locations as the used sample for the third order, we only tested increasing available sample sizes ranging from 10 to 50 per used in increments of 10 for the third order. For both orders, we used the greatest number of required locations of any season for all seasons.

## 3. Results

Our elk range sample size varied by season. Late gestation had a sample size of 63 ranges from 32 cows, calving season had 61 ranges from 31 cows, breeding season had 57 ranges from 30 cows, and mid-gestation had 69 ranges from 29 cows. Our sensitivity analyses determined the need for 1500 random used and 30,000 random available locations (20 per used) for each individual at the second order and 20 random available locations per used location at the third order (see Appendix A, Table A1, Table A2, Table A3, Table A4, Table A5, Table A6, Table A7, Table A8, Table A9, Table A10, Table A11 and Table A12). Slope was excluded from all models due to collinearity with terrain roughness, which generally had greater explanatory power (|r| = 0.976 for all seasons at the second order, and 0.977 during late gestation, breeding, and mid-gestation and 0.976 during calving at the third order). The global model was the top model for every season. There were no competing models at either order of analyses or during any season (delta AICc > 4; see Appendix A, Table A13, Table A14, Table A15, Table A16, Table A17, Table A18, Table A19, Table A20, Table A21, Table A22, Table A23, Table A24, Table A25, Table A26, Table A27 and Table A28).

**Figure 2:**
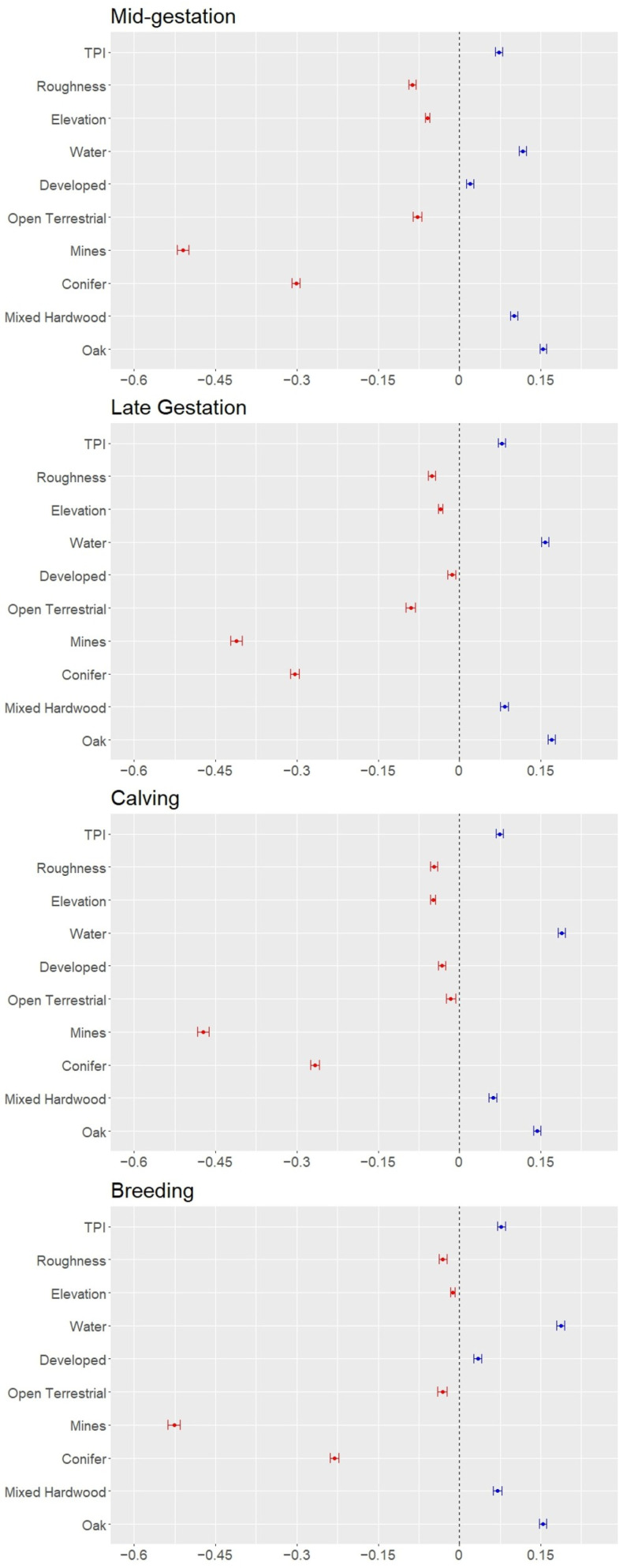
Beta coefficients for selection at the second order during the mid-gestational (December–February), late gestational (March–May), calving (June–August), and breeding (September–November) biological seasons for adult female elk in southwestern Virginia from data collected January 2019 through November 2022. Land cover types and terrain variables are on the *y*-axis with beta coefficient values on the *x*-axis. Variables to the left of 0 (red) indicate selection of areas closer to the land cover type or lower terrain values. Variables to the right of 0 (blue) indicate selection of areas farther from land cover types and higher terrain values. Variables are in no particular order.

**Figure 3:**
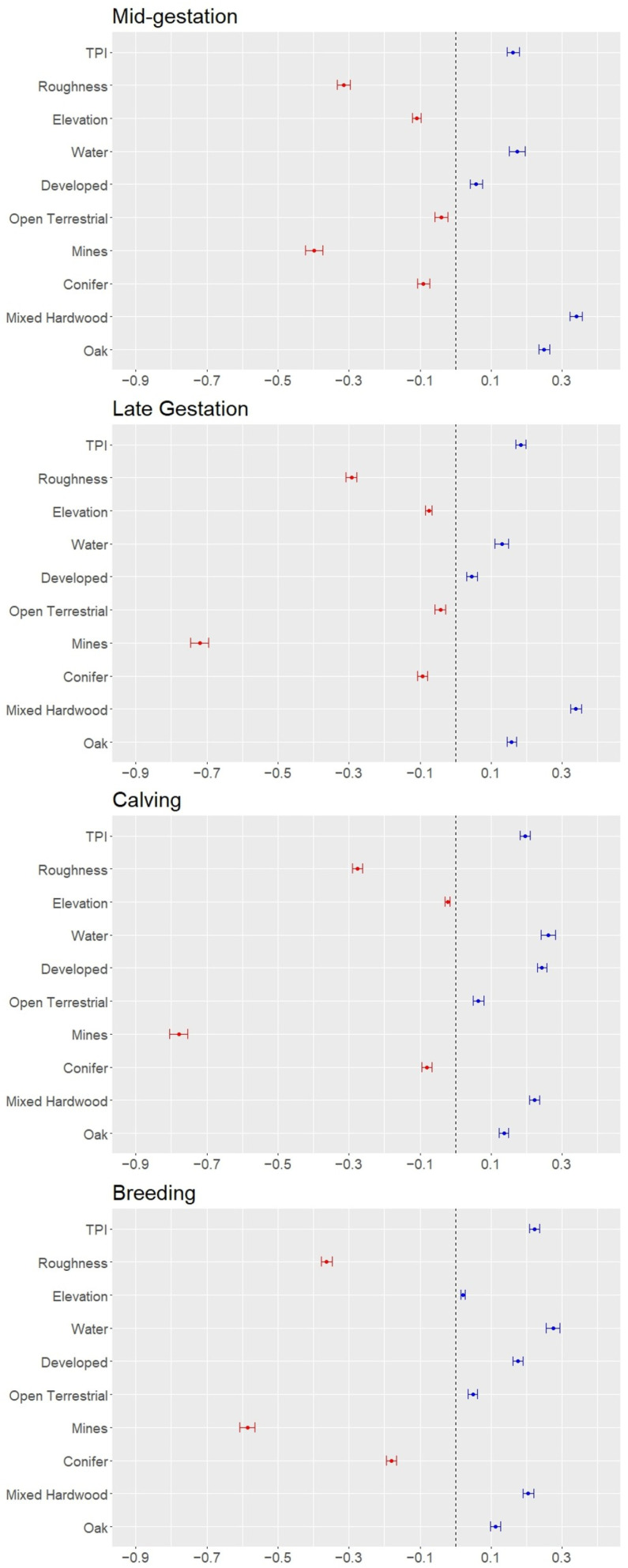
Beta coefficients for selection at the third order during the mid-gestational (December–February), late gestational (March–May), calving (June–August), and breeding (September–November) biological seasons for adult female elk in southwestern Virginia from data collected January 2019 through November 2022. Land cover types and terrain variables are on the *y*-axis with beta coefficient values on the *x*-axis. Variables to the left of 0 (red) indicate selection of areas closer to the land cover type or lower terrain values. Variables to the right of 0 (blue) indicate selection of areas farther from land cover types and higher terrain values. Variables are in no particular order.

During every season, and at both orders, female elk selected for mined lands, conifer forests, ridge tops, and lower terrain roughness but avoided oak and mixed hardwood forests, human development, and water (Figure 2 and Figure 3; Appendix A, Table A29 and Table A30). Further, female elk selected moderate elevations during all seasons at both orders except for during the breeding season at the third order wherein female elk avoided moderate elevations and opted for high or relatively lower elevations (Figure 2 and Figure 3; Appendix A, Table A29 and Table A30). Unlike the coal mines which were selected at both orders throughout the year, unmined open lands were selected during all seasons at the second order but only selected at the third order during mid- and late gestation (Figure 2 and Figure 3; Appendix A, Table A29 and Table A30). Female elk selected developed areas during the late gestation and calving periods at the second order but avoided them throughout the year at the third order (Figure 2 and Figure 3; Appendix A, Table A29 and Table A30). At both the second and third order, the strongest selection was for mines, but the strongest avoidance of other habitat types varied by order and season.

At the second order, the influence of land cover and topographic variables were generally consistent across seasons. For the distance-based approach, negative beta coefficients indicate selection of areas closer to the variable whereas positive values indicate selection of areas farther from the variable (i.e., avoidance). Female elk most strongly selected areas closer to mines (β = −0.411 SE = ±0.005 during late gestation, −0.473 ± 0.005 during calving, −0.526 ± 0.006 during breeding, and −0.510 ± 0.005 during mid-gestation) followed by areas closer to conifer forests (β = −0.304 SE = ±0.004 during late gestation, −0.266 ± 0.004 during calving, −0.231 ± 0.004 during breeding, and −0.301 ± 0.004 during mid-gestation; Figure 2). Further, late gestation, breeding, and mid-gestation had the same top five explanatory variables, adding distances to oak forests (β = 0.170 SE = ±0.003 during late gestation, 0.153 ± 0.003 during breeding, and 0.154 ± 0.003 during mid-gestation), water (β = 0.158 ± 0.003 during late gestation, 0.187 ± 0.004 during breeding, and 0.116 ± 0.003 during mid-gestation), and mixed hardwood forests (β = 0.083 SE = ±0.004 during late gestation, 0.070 ± 0.004 during breeding, and 0.100 ± 0.003 during mid-gestation; Figure 2). During calving season, female elk also selected areas farther from water (β = 0.189 SE = ±0.003) and oak forests (0.143 ± 0.003) but switched to areas farther from mixed hardwood forests with selection of moderate elevations (elevation^2^, β = −0.049 SE = ±0.002; Figure 2). Overall, elevation, TPI, terrain roughness, and distances to unmined open land and developed areas influenced seasonal selection at different magnitudes across seasons (Appendix A, Table A29). Topographic variables (elevation, terrain roughness, and TPI) largely had less explanatory power than land cover types on resource selection at the second order (Appendix A, Table A29). However, distances to development and unmined open land were generally the least important land cover variables across the year, and in many cases, less contributory than the topographic variables (Appendix A, Table A29).

Third order selection largely had differing explanatory variable ranks amongst seasons relative to the second order (Appendix A, Table A29 and Table A30). During each season, female elk strongly selected areas closer to mines (β = −0.720 SE = ±0.013 during late gestation, −0.779 ± 0.013 during calving, −0.586 ± 0.011 during breeding, and 0.397 ± 0.012 during mid-gestation), areas farther from mixed hardwood forests (β = 0.339 SE = ±0.008 during late gestation, 0.223 ± 0.007 during calving, 0.205 ± 0.008 during breeding, and 0.340 ± 0.009 during mid-gestation), and reduced topographic roughness (β = −0.293 SE = ±0.008 during late gestation, −0.276 ± 0.008 during calving, −0.363 ± 0.008 during breeding, and −0.315 ± 0.009 during mid-gestation) as the top three variables, but they varied by order of explanatory power (Figure 3; Appendix A, Table A30). Distance to unmined open land was consistently one of the least influential variables during all seasons (β = −0.042 SE = ±0.008 during late gestation, 0.064 ± 0.008 during calving, 0.049 ± 0.007 during breeding, and −0.040 ± 0.010 during mid-gestation; Figure 3; Appendix A, Table A30). Although topographic variables were more important at the third order than the second order, elevation was consistently in the bottom half of order of influence on selection and was the least important variable during calving (β = −0.023 SE = ±0.003) and breeding (0.021 ± 0.003) seasons (Figure 3; Appendix A, Table A30). We observed limited signal switching (changes in the direction of the effect of a resource on the probability of its use) from our second order analysis to our third order analysis. Developed areas were slightly selected at the second order during late gestation (β = −0.014 SE = ±0.004) and calving (−0.033 ± 0.004) but switched to slightly avoided during late gestation (0.046 ± 0.007) and strongly avoided during calving seasons (0.244 ± 0.007) at the third order (Figure 2 and Figure 3; Appendix A, Table A29 and Table A30). Additionally, unmined open land was selected during all seasons at the second order but were avoided at the third order during calving and breeding seasons (Figure 2 and Figure 3; Appendix A, Table A29 and Table A30).

Goodness-of-fit and predictive power of all models was evaluated using k-fold cross validation (see Appendix A, Table A31 and Table A32). Top models at both orders performed well (i.e., >0.8; see Appendix A, Table A31 and Table A32). At the second order, mid-gestation had an observed mean of 1.0000 (Std. Dev. = 0.0000), late gestation had an observed mean of 1.0000 (0.0000), calving season had an observed mean of 1.0000 (0.0000), and breeding season had an observed mean of 0.9999 (0.0012). At the third order, mid-gestation had an observed mean of 0.9400 (Std. Dev. = 0.0233), late gestation had an observed mean of 0.9328 (0.0258), calving season had an observed mean of 0.9842 (0.0111), and breeding season had an observed mean of 0.9767 (0.0189).

## 4. Discussion

After reintroduction and successful herd establishment in the VEMZ, we observed seasonal variation in magnitude of selection and avoidance for both land cover and topographic variables across orders of selection. Female elk strongly selected reclaimed mines throughout the year at both the second and third orders likely for both forage and cover resources. Surprisingly, whereas unmined open land was selected throughout the year at the second order, it was only selected during mid- and late gestation at the third order. Based on previous research in neighboring elk populations [24,26], we predicted that female elk would select forested land cover during spring (late gestation) and breeding (fall) to take advantage of high-quality ephemeral forage resources such as new plant growth and hard mast. In contrast, female elk avoided deciduous forests (both oak and mixed hardwood), the dominant land cover in our study area, throughout the year at both orders. But as expected, female elk prioritized lower terrain roughness along ridgetops at moderate elevations possibly to ease movements across the rugged landscape. Generally, land cover types were more important for female elk at the second order with increasing importance of terrain variables at the third order. This reflects how ungulate seasonal and home range establishment is largely dictated by the general “foodscape” and landscape-scale predation risk, whereas competition, cover, and limited high-quality forage has a greater influence on movements within the range [8,17].

We found female elk consistently selected moderate elevations, ridgetops, and lower terrain roughness with little change between seasons. Overall, topographic variables had greater influence over selection at the third order than the second order. Of the topographic variables, terrain roughness had the greatest impact on selection, while elevation had the least impact. Because our study area was extremely rugged where unmined, gentler topography was invariably important for these elk, as has been previously described in neighboring populations [16,34]. However, compared to elk in western North America that utilize more rugged terrain to potentially decrease predation risk, rugged terrain appears to be much less important for elk in the eastern North America, likely because large carnivores are mostly absent [12,13,17]. Although we did not find a correlation between mines and TPI, surface coal mines are generally associated with flatter ridgetops. Female elk will feed on reclaimed mines, then may rest and ruminate along finger ridges to maintain vigilance without moving down into ravines as observed in other elk populations in eastern [71] and western North America [72] and as observed in other cervids [73,74,75]. In contrast to western North America, elevational shifts during different times of the year were not observed in our population [31,76,77]. This is likely due to milder winters and less snowfall, which does not necessitate tracking spatiotemporal gradients in forage availability that propagate across elevational and latitudinal gradients [32,78,79].

As expected, mines were the highest selected variable at both orders of our analysis. In our largely forested landscape, reclaimed mines provide year-round foraging resources for elk, primarily in the form of graminoids and legumes [24,26]. Reclaimed mines are not exclusively grasslands and often have dense edges where they meet forests [41,42,43]. These areas of vegetation may provide additional forage and cover resources important to elk such as thermal refugia and escape cover [15,72,80]. Thus, our results further support other findings from the Coalfields and elsewhere where elk select reclaimed mines [71,81].

Female elk avoided both deciduous forest types (mixed-hardwood and oak) at both orders throughout the year. The Intermediate Disturbance Hypothesis proposes biodiversity is highest in areas with returning disturbance intervals [82], which from a forage quality perspective, should benefit elk. However, since the large-scale logging of the Appalachian Mountains during the late 19th and early 20th centuries, continued poor forest management in the Coalfields has resulted in lower vegetation diversity with little-to-no herbaceous ground cover [83,84,85]. In the Coalfields, surface coal mining is the main form of disturbance [41,86], but by virtue, does not have a return interval. The remaining forested landscape often does not experience regular returning disturbance intervals (i.e., prescribed fire or forest harvesting with regard for regeneration) that are beneficial to many wildlife species [85,87,88]. If the herbaceous layer is present in these forests, it is often dominated by low forage quality ericaceous shrubs such as rhododendrons and mountain laurel or exotic invasives [42,48,84]. However, female elk showed the greatest tolerance of deciduous forests during the breeding and calving seasons during which times deciduous forests may provide limited durations of high forage availability, calf-rearing cover, and possible hot-weather thermal refugia [22,26,89,90]. Conversely, we found deciduous forests were most strongly avoided during the winter (mid-gestation) leaf-off period due to little browse availability and limited cover as observed for elk in eastern North America by other researchers [24,26,91].

Surprisingly, female elk selected for conifer forests at both orders during all seasons. Cervids prioritize habitat diversity, heterogeneity, and forest structure [92,93,94], which may be magnified due to their proximity to reclaimed mines [41,43]. Elk in western North American populations utilize conifers for thermal cover [14,44] and to avoid predation [11]. Comparatively, with mild winters in the Coalfields, elk may not need to rely on conifer forests for cold thermal refugia. However, thermal cover may be more critical in warmer temperatures. With elk being large-bodied endotherms, the Heat Dissipation Limit Theory indicates animals will rest to dissipate metabolic heat [95]. During the summer, moose (*Alces alces*) will seek coniferous forests during the day as temperature increases [15], and elk have shown a propensity for conifer stands during the summer in other populations [31]. Conversely, during the calving season when temperatures in our study area are at or near their warmest, selection for conifer forests was the weakest. Additionally, as mentioned, adult elk in our study area do not face the same predation pressure as western North American populations due to a lack of large predators such as brown bear (*Ursus arctos*), mountain lions (*Felis concolor*), or wolves (*Canis lupus*; [96,97]); however, the “Landscape of Fear” [98] may persist through evolved psychological mechanisms such as the Baldwin Effect [99], predation on cervid neonates by black bear (*Ursus americanus*) [100], or human activity.

Developed areas (i.e., anthropogenic infrastructure) may provide some forage resources in the form of edge habitat [8,92,101], but frequent or perceived human activity in developed areas may deter their use [8,31,102]. We found support for this as female elk largely avoided developed areas in our study area at both orders throughout the year. However, selection of developed areas during late gestation and calving at the second order could be an artifact of our habitat type classification. Where hunted or where road density and traffic are high, elk tend to avoid people and developed areas [8,103,104]. We found support for this as female elk largely avoided developed areas. In addition to greater anthropogenic infrastructure, developed areas in our study area included many secondary, dirt roads on restricted access mines and natural gas wells with limited day-to-day human use. For elk, these secondary roads may provide easier travel across the landscape and long linear corridors of day-lighted edge habitat with abundant forbs, early-successional woody browse, and dense escape cover [8,81,92]. In our system, secondary roadsides are often planted in seed mixes that include species selected for erosion control but that are also quality forage (e.g., *Rubus* spp., *Trifolium* spp., ryegrass, and orchard grass). At the second order during late gestation and calving when metabolic requirements are at their highest [23,76], and when female elk and their offspring are most vulnerable to predation [17,105], female elk selected developed areas. Selection of developed areas during this time of year may stem from the avoidance of such areas by large carnivores–a concept known as the “human shield” hypothesis [8,106,107]. However, female elk strongly avoided developed areas at the third order during calving season when forage and cover availability would be at their yearly highest. Although our developed land cover type did include large amounts of secondary roads, it also included human communities, infrastructure, and housing. These heavy human-activity features of our developed habitat type may have overshadowed the benefits provided by secondary roads at the third order.

The unmined open land cover type, which conceivably provides similar higher quality forage opportunities as reclaimed mines, was only selected during mid- and late gestation at the third order (Figure 3). The unmined open land type included areas such as improved pasture, but also small (<5 ha), managed wildlife openings which provide elk with high-quality forage in the VEMZ. However, at the third order, female elk only selected unmined open land during times when forage availability is lowest on the landscape (mid-gestation; [108]) and when female elk have increasing metabolic requirements associated with gestation (late gestation; [23,76,108]). Beale and Boyce [81] found similar results on reclaimed mines in Alberta, Canada, where elk heavily selected reclaimed grasslands, but avoided other grassland types. Certainly, the selection of unmined open land devoted to livestock or hay production (the majority of unmined open lands in our study area) likely increases the possibility human-wildlife conflict [109] or other factors not easily quantified, such as a constant presence of domestic dogs (*Canis familiarus*). Further, elk have been observed to avoid domestic cattle [10] possibly due to their large dietary overlap [9]. However, female elk may have greater tolerance of domestic cattle during mid-gestation and calving, when forage resources are less abundant, and during calving, because of increased metabolic demands, respectfully.

## 5. Conclusions

Reintroductions provide a means of returning species to their native ranges following extirpation. Elk have a long history of reintroduction efforts spanning the past century [110,111,112]. Over the past four decades, multi-state initiatives have successfully restored elk across the central and eastern North America. In the Appalachian Mountains, reintroductions have been focused on open landscapes, specifically reclaimed surface mines [45,112]. Open habitats are important resources for elk across their range [38,77,92,113]; however, we established that elk do not perceive all open habitats as equal, which must be considered for future reintroductions. Elk reintroductions in the Coalfields found success with reclaimed surface coal mines [45,112] which largely persist in states of arrested succession [41], offering greater structural and species diversity when compared to other open habitats in our study area (e.g., cattle pasture; [41,43]). Our results showed reclaimed mines were important for female elk throughout the year at the second and third order as they likely provide both high quality forage and cover. These restored patches were a small portion of our study area’s landscape within an expanse of largely neglected forested ecosystem. Although our analyses were limited to female elk, we believe our results were generally applicable to males as well based on similar results from male elk in nearby populations [16,24,38]. With the decline of the coal industry [49,86], other forms of forest disturbance and the creation and maintenance of open areas will become increasingly important for wildlife in the future.

## Data Availability

The datasets generated during and/or analyzed during the current study are available from the corresponding author upon reasonable request.

## Abbreviations

The following abbreviations are used in this manuscript:

VDWR	Virginia Department of Wildlife Resources
VEMZ	Virginia Elk Management Zone
GPS	Global Positioning System
AKDE	Autocorrelated Kernel Density Estimate
GAP	National Gap Analysis Project
NTH	Northeastern Terrestrial Habitats
DEM	Digital Elevation Model
TPI	Topographic Position Index
VIF	Variance Inflation Factors
AICc	Akaike Information Criterion corrected for small sample size

## Appendix A

**Table A1:** Results from the sensitivity analysis for varying random used sample size during the mid-gestation biological season (December–February) at the second order for adult female elk in southwestern Virginia from data collected January 2019 through November 2022. The available sample size was held constant at 20,000 locations.

	Number Random Used Points—20,000 Available							
Full Model	500 Points		1000 Points		1500 Points		2000 Points	
Term *	Estimate	Std. Error	Estimate	Std. Error	Estimate	Std. Error	Estimate	Std. Error
(Intercept)	−10.7902	0.0397	−10.0767	0.0374	−9.6544	0.0361	−9.3604	0.0365
s_TPI	0.0782	0.0058	0.0743	0.0041	0.0711	0.0033	0.0720	0.0029
s_rough	−0.0921	0.0058	−0.0843	0.0041	−0.0872	0.0034	−0.0906	0.0029
I(s_elevation^2^)	−0.0491	0.0034	−0.0560	0.0025	−0.0626	0.0021	−0.0636	0.0018
s_distoak	0.1580	0.0052	0.1535	0.0037	0.1561	0.0030	0.1556	0.0026
s_distmh	0.0941	0.0059	0.0980	0.0041	0.0995	0.0034	0.1025	0.0029
s_distcon	−0.3150	0.0064	−0.3058	0.0045	−0.2993	0.0036	−0.2955	0.0031
s_distot	−0.0706	0.0072	−0.0724	0.0051	−0.0793	0.0041	−0.0758	0.0036
s_distmine	−0.5161	0.0092	−0.5148	0.0064	−0.5088	0.0052	−0.5131	0.0045
s_distdev	0.0219	0.0058	0.0205	0.0041	0.0200	0.0033	0.0192	0.0029
s_distwater	0.1200	0.0057	0.1190	0.0040	0.1152	0.0033	0.1144	0.0028

* “s_” indicates variable was scaled and centered.

**Table A2:** Results from the sensitivity analysis for varying random used sample size during the late gestation biological season (March–May) at the second order for adult female elk in southwestern Virginia from data collected January 2019 through November 2022. The available sample size was held constant at 20,000 locations.

	Number Random Used Points—20,000 Available							
Full Model	500 Points		1000 Points		1500 Points		2000 Points	
Term *	Estimate	Std. Error	Estimate	Std. Error	Estimate	Std. Error	Estimate	Std. Error
(Intercept)	−10.7488	0.0322	−10.0503	0.0324	−9.6354	0.0313	−9.3359	0.0305
s_TPI	0.0755	0.0060	0.0772	0.0043	0.0806	0.0035	0.0810	0.0030
s_rough	−0.0465	0.0060	−0.0478	0.0043	−0.0503	0.0035	−0.0514	0.0030
I(s_elevation^2^)	−0.0354	0.0034	−0.0345	0.0024	−0.0360	0.0020	−0.0395	0.0017
s_distoak	0.1665	0.0054	0.1699	0.0038	0.1735	0.0032	0.1757	0.0028
s_distmh	0.0892	0.0062	0.0861	0.0044	0.0800	0.0036	0.0831	0.0031
s_distcon	−0.2970	0.0066	−0.3048	0.0047	−0.3042	0.0038	−0.2993	0.0033
s_distot	−0.0912	0.0077	−0.0888	0.0054	−0.0895	0.0044	−0.0942	0.0038
s_distmine	−0.4174	0.0092	−0.4153	0.0065	−0.4107	0.0053	−0.4062	0.0045
s_distdev	−0.0192	0.0063	−0.0144	0.0044	−0.0142	0.0036	−0.0114	0.0031
s_distwater	0.1595	0.0058	0.1598	0.0041	0.1578	0.0033	0.1572	0.0029

* “s_” indicates variable was scaled and centered.

**Table A3:** Results from the sensitivity analysis for varying random used sample size during the calving biological season (June–August) at the second order for adult female elk in southwestern Virginia from data collected January 2019 through November 2022. The available sample size was held constant at 20,000 locations.

	Number Random Used Points—20,000 Available							
Full Model	500 Points		1000 Points		1500 Points		2000 Points	
Term *	Estimate	Std. Error	Estimate	Std. Error	Estimate	Std. Error	Estimate	Std. Error
(Intercept)	−10.7157	0.0227	−10.0223	0.0245	−9.6080	0.0240	−9.3094	0.0232
s_TPI	0.0740	0.0061	0.0729	0.0043	0.0737	0.0035	0.0732	0.0031
s_rough	−0.0476	0.0062	−0.0502	0.0044	−0.0470	0.0036	−0.0480	0.0031
I(s_elevation^2^)	−0.0509	0.0037	−0.0472	0.0026	−0.0502	0.0021	−0.0542	0.0019
s_distoak	0.1433	0.0056	0.1439	0.0040	0.1464	0.0033	0.1491	0.0028
s_distmh	0.0602	0.0065	0.0606	0.0046	0.0617	0.0037	0.0592	0.0032
s_distcon	−0.2550	0.0067	−0.2672	0.0047	−0.2679	0.0039	−0.2695	0.0033
s_distot	−0.0176	0.0074	−0.0148	0.0052	−0.0188	0.0043	−0.0186	0.0037
s_distmine	−0.4760	0.0094	−0.4728	0.0066	−0.4695	0.0054	−0.4677	0.0046
s_distdev	−0.0280	0.0064	−0.0289	0.0045	−0.0309	0.0037	−0.0331	0.0032
s_distwater	0.1939	0.0059	0.1902	0.0042	0.1874	0.0034	0.1854	0.0029

* “s_” indicates variable was scaled and centered.

**Table A4:** Results from the sensitivity analysis for varying random used sample size during the breeding biological season (September–November) at the second order for adult female elk in southwestern Virginia from data collected January 2019 through November 2022. The available sample size was held constant at 20,000 locations.

	Number Random Used Points—20,000 Available							
Full Model	500 Points		1000 Points		1500 Points		2000 Points	
Term *	Estimate	Std. Error	Estimate	Std. Error	Estimate	Std. Error	Estimate	Std. Error
(Intercept)	−10.7679	0.0345	−10.0656	0.0344	−9.6562	0.0352	−9.3600	0.0352
s_TPI	0.0794	0.0063	0.0770	0.0044	0.0787	0.0036	0.0811	0.0031
s_rough	−0.0282	0.0063	−0.0293	0.0045	−0.0286	0.0036	−0.0287	0.0032
I(s_elevation^2^)	−0.0125	0.0033	−0.0136	0.0023	−0.0154	0.0019	−0.0171	0.0017
s_distoak	0.1488	0.0058	0.1530	0.0041	0.1544	0.0034	0.1539	0.0029
s_distmh	0.0646	0.0067	0.0662	0.0047	0.0725	0.0039	0.0736	0.0034
s_distcon	−0.2391	0.0069	−0.2290	0.0048	−0.2287	0.0039	−0.2282	0.0034
s_distot	−0.0406	0.0078	−0.0365	0.0055	−0.0324	0.0045	−0.0265	0.0039
s_distmine	−0.5159	0.0100	−0.5158	0.0070	−0.5261	0.0057	−0.5287	0.0049
s_distdev	0.0321	0.0064	0.0338	0.0045	0.0348	0.0037	0.0354	0.0032
s_distwater	0.1831	0.0062	0.1837	0.0044	0.1847	0.0035	0.1824	0.0030

* “s_” indicates variable was scaled and centered.

**Table A5:** Results from the sensitivity analysis for varying random available sample size during the mid-gestation biological season (December–February) at the second order for adult female elk in southwestern Virginia from data collected January 2019 through November 2022. The used sample size was held constant at 1500 locations and available locations increased iteratively by 10 from 10 points per used to 50 points per used.

	Number Random Available Points per Used—1500 Used									
Full Model	10 Points		20 Points		30 Points		40 Points		50 Points	
Term *	Estimate	Std. Error	Estimate	Std. Error	Estimate	Std. Error	Estimate	Std. Error	Estimate	Std. Error
(Intercept)	−9.3562	0.0358	−10.0730	0.0370	−10.4864	0.0368	−10.7787	0.0369	−11.0049	0.0366
s_TPI	0.0743	0.0033	0.0729	0.0033	0.0725	0.0033	0.0713	0.0033	0.0731	0.0033
s_rough	−0.0888	0.0034	−0.0871	0.0034	−0.0886	0.0034	−0.0896	0.0034	−0.0889	0.0034
I(s_elevation^2^)	−0.0649	0.0021	−0.0588	0.0021	−0.0567	0.0020	−0.0555	0.0020	−0.0548	0.0020
s_distoak	0.1595	0.0031	0.1543	0.0030	0.1536	0.0030	0.1507	0.0030	0.1523	0.0030
s_distmh	0.0982	0.0034	0.1003	0.0034	0.0981	0.0034	0.0998	0.0034	0.0998	0.0034
s_distcon	−0.2970	0.0036	−0.3014	0.0036	−0.3005	0.0036	−0.3013	0.0037	−0.3023	0.0037
s_distot	−0.0784	0.0042	−0.0774	0.0042	−0.0779	0.0042	−0.0796	0.0042	−0.0787	0.0042
s_distmine	−0.5061	0.0052	−0.5097	0.0053	−0.5119	0.0053	−0.5122	0.0053	−0.5131	0.0053
s_distdev	0.0201	0.0033	0.0199	0.0033	0.0204	0.0033	0.0206	0.0034	0.0199	0.0034
s_distwater	0.1148	0.0033	0.1163	0.0033	0.1171	0.0033	0.1175	0.0033	0.1165	0.0033

* “s_” indicates variable was scaled and centered.

**Table A6:** Results from the sensitivity analysis for varying random available sample size during the late gestation biological season (March–May) at the second order for adult female elk in southwestern Virginia from data collected January 2019 through November 2022. The used sample size was held constant at 1500 locations and available locations increased iteratively by 10 from 10 points per used to 50 points per used.

	Number Random Available Points per Used—1500 Used									
Full Model	10 Points		20 Points		30 Points		40 Points		50 Points	
Term *	Estimate	Std. Error	Estimate	Std. Error	Estimate	Std. Error	Estimate	Std. Error	Estimate	Std. Error
(Intercept)	−9.3359	0.0310	−10.0483	0.0316	−10.4610	0.0316	−10.7529	0.0319	−10.9778	0.0316
s_TPI	0.0794	0.0035	0.0782	0.0035	0.0797	0.0035	0.0813	0.0035	0.0798	0.0035
s_rough	−0.0485	0.0035	−0.0507	0.0035	−0.0506	0.0035	−0.0504	0.0035	−0.0516	0.0035
I(s_elevation^2^)	−0.0397	0.0020	−0.0349	0.0020	−0.0326	0.0020	−0.0314	0.0019	−0.0314	0.0019
s_distoak	0.1752	0.0032	0.1699	0.0031	0.1702	0.0031	0.1704	0.0031	0.1691	0.0031
s_distmh	0.0832	0.0036	0.0828	0.0036	0.0808	0.0036	0.0807	0.0036	0.0809	0.0036
s_distcon	−0.3011	0.0038	−0.3039	0.0038	−0.3042	0.0038	−0.3047	0.0038	−0.3060	0.0038
s_distot	−0.0915	0.0044	−0.0897	0.0044	−0.0903	0.0044	−0.0913	0.0044	−0.0898	0.0044
s_distmine	−0.4062	0.0052	−0.4113	0.0053	−0.4114	0.0053	−0.4115	0.0053	−0.4124	0.0053
s_distdev	−0.0143	0.0036	−0.0140	0.0036	−0.0138	0.0036	−0.0152	0.0036	−0.0151	0.0036
s_distwater	0.1559	0.0033	0.1575	0.0033	0.1580	0.0034	0.1588	0.0034	0.1588	0.0034

* “s_” indicates variable was scaled and centered.

**Table A7:** Results from the sensitivity analysis for varying random available sample size during the calving biological season (June–August) at the second order for adult female elk in southwestern Virginia from data collected January 2019 through November 2022. The used sample size was held constant at 1500 locations and available locations increased iteratively by 10 from 10 points per used to 50 points per used.

	Number Random Available Points per Used—1500 Used									
Full Model	10 Points		20 Points		30 Points		40 Points		50 Points	
Term *	Estimate	Std. Error	Estimate	Std. Error	Estimate	Std. Error	Estimate	Std. Error	Estimate	Std. Error
(Intercept)	−9.3100	0.0232	−10.0200	0.0240	−10.4347	0.0246	−10.7262	0.0250	−10.9506	0.0248
s_TPI	0.0714	0.0035	0.0740	0.0035	0.0749	0.0035	0.0744	0.0035	0.0742	0.0035
s_rough	−0.0462	0.0035	−0.0471	0.0036	−0.0470	0.0036	−0.0484	0.0036	−0.0484	0.0036
I(s_elevation^2^)	−0.0537	0.0021	−0.0489	0.0021	−0.0459	0.0021	−0.0448	0.0021	−0.0449	0.0021
s_distoak	0.1455	0.0033	0.1430	0.0033	0.1440	0.0032	0.1426	0.0033	0.1428	0.0032
s_distmh	0.0628	0.0037	0.0618	0.0037	0.0612	0.0037	0.0624	0.0037	0.0608	0.0037
s_distcon	−0.2654	0.0038	−0.2664	0.0039	−0.2686	0.0039	−0.2693	0.0039	−0.2687	0.0039
s_distot	−0.0199	0.0043	−0.0158	0.0043	−0.0183	0.0043	−0.0180	0.0043	−0.0183	0.0043
s_distmine	−0.4710	0.0054	−0.4731	0.0054	−0.4732	0.0054	−0.4738	0.0054	−0.4747	0.0054
s_distdev	−0.0310	0.0037	−0.0324	0.0037	−0.0301	0.0037	−0.0318	0.0037	−0.0310	0.0037
s_distwater	0.1868	0.0034	0.1890	0.0034	0.1886	0.0034	0.1892	0.0034	0.1891	0.0034

* “s_” indicates variable was scaled and centered.

**Table A8:** Results from the sensitivity analysis for varying random available sample size during the breeding biological season (September–November) at the second order for adult female elk in southwestern Virginia from data collected January 2019 through November 2022. The used sample size was held constant at 1500 locations and available locations increased iteratively by 10 from 10 points per used to 50 points per used.

	Number Random Available Points per Used—1500 Used									
Full Model	10 Points		20 Points		30 Points		40 Points		50 Points	
Term *	Estimate	Std. Error	Estimate	Std. Error	Estimate	Std. Error	Estimate	Std. Error	Estimate	Std. Error
(Intercept)	−9.3614	0.0351	−10.0715	0.0357	−10.4846	0.0359	−10.7765	0.0361	−11.0007	0.0364
s_TPI	0.0792	0.0036	0.0773	0.0036	0.0780	0.0036	0.0787	0.0036	0.0779	0.0036
s_rough	−0.0306	0.0036	−0.0305	0.0036	−0.0306	0.0036	−0.0312	0.0036	−0.0311	0.0036
I(s_elevation^2^)	−0.0164	0.0019	−0.0124	0.0019	−0.0113	0.0019	−0.0101	0.0019	−0.0101	0.0019
s_distoak	0.1572	0.0034	0.1534	0.0033	0.1524	0.0033	0.1527	0.0033	0.1514	0.0033
s_distmh	0.0723	0.0039	0.0703	0.0038	0.0711	0.0038	0.0710	0.0038	0.0724	0.0038
s_distcon	−0.2293	0.0039	−0.2307	0.0040	−0.2325	0.0040	−0.2309	0.0040	−0.2313	0.0040
s_distot	−0.0288	0.0045	−0.0314	0.0045	−0.0306	0.0045	−0.0308	0.0045	−0.0315	0.0045
s_distmine	−0.5246	0.0057	−0.5263	0.0057	−0.5305	0.0058	−0.5316	0.0058	−0.5307	0.0058
s_distdev	0.0329	0.0037	0.0340	0.0037	0.0336	0.0037	0.0332	0.0037	0.0339	0.0037
s_distwater	0.1849	0.0035	0.1866	0.0036	0.1854	0.0036	0.1888	0.0036	0.1878	0.0036

* “s_” indicates variable was scaled and centered.

**Table A9:** Results from the sensitivity analysis for varying random available sample size during the mid-gestation biological season (December–February) at the third order for adult female elk in southwestern Virginia from data collected January 2019 through November 2022. The used sample size was held constant at the number of true locations collected from the elk and available locations increased iteratively by 10 from 10 points per used to 50 points per used.

	Number Random Available Points per Real									
Full Model	10 Points		20 Points		30 Points		40 Points		50 Points	
Term *	Estimate	Std. Error	Estimate	Std. Error	Estimate	Std. Error	Estimate	Std. Error	Estimate	Std. Error
(Intercept)	−9.5084	0.0935	−10.2416	0.0914	−10.6562	0.0901	−10.9512	0.0897	−11.1769	0.0896
s_TPI	0.1583	0.0088	0.1624	0.0088	0.1642	0.0088	0.1690	0.0088	0.1673	0.0088
s_rough	−0.3233	0.0092	−0.3150	0.0092	−0.3225	0.0092	−0.3221	0.0092	−0.3187	0.0092
I(s_elevation^2^)	−0.1194	0.0064	−0.1082	0.0062	−0.1097	0.0063	−0.1069	0.0062	−0.1076	0.0063
s_distoak	0.2615	0.0080	0.2493	0.0077	0.2558	0.0077	0.2520	0.0077	0.2493	0.0076
s_distmh	0.3410	0.0093	0.3401	0.0089	0.3304	0.0089	0.3280	0.0089	0.3290	0.0088
s_distcon	−0.0925	0.0086	−0.0900	0.0087	−0.0863	0.0087	−0.0946	0.0087	−0.0916	0.0088
s_distot	−0.0288	0.0096	−0.0398	0.0097	−0.0340	0.0097	−0.0355	0.0098	−0.0376	0.0098
s_distmine	−0.3982	0.0124	−0.3971	0.0125	−0.3949	0.0125	−0.3973	0.0125	−0.3947	0.0125
s_distdev	0.0535	0.0088	0.0585	0.0088	0.0598	0.0088	0.0592	0.0089	0.0625	0.0089
s_distwater	0.1757	0.0116	0.1737	0.0116	0.1692	0.0117	0.1750	0.0116	0.1737	0.0117

* “s_” indicates variable was scaled and centered.

**Table A10:** Results from the sensitivity analysis for varying random available sample size during the late gestation biological season (March–May) at the third order for adult female elk in southwestern Virginia from data collected January 2019 through November 2022. The used sample size was held constant at the number of true locations collected from the elk and available locations increased iteratively by 10 from 10 points per used to 50 points per used.

	Number Random Available Points per Real									
Full Model	10 Points		20 Points		30 Points		40 Points		50 Points	
Term *	Estimate	Std. Error	Estimate	Std. Error	Estimate	Std. Error	Estimate	Std. Error	Estimate	Std. Error
(Intercept)	−9.5316	0.0846	−10.2702	0.0854	−10.6907	0.0858	−10.9833	0.0848	−11.2105	0.0840
s_TPI	0.1794	0.0073	0.1829	0.0073	0.1810	0.0073	0.1788	0.0073	0.1847	0.0073
s_rough	−0.2965	0.0077	−0.2932	0.0076	−0.2985	0.0077	−0.2967	0.0076	−0.2959	0.0076
I(s_elevation^2^)	−0.0852	0.0047	−0.0752	0.0046	−0.0763	0.0046	−0.0743	0.0046	−0.0732	0.0046
s_distoak	0.1602	0.0070	0.1579	0.0068	0.1587	0.0067	0.1553	0.0068	0.1510	0.0067
s_distmh	0.3318	0.0078	0.3391	0.0077	0.3348	0.0076	0.3319	0.0076	0.3323	0.0076
s_distcon	−0.0915	0.0072	−0.0928	0.0073	−0.0950	0.0073	−0.0929	0.0073	−0.0912	0.0073
s_distot	−0.0416	0.0079	−0.0423	0.0080	−0.0418	0.0080	−0.0439	0.0080	−0.0422	0.0081
s_distmine	−0.7296	0.0132	−0.7201	0.0132	−0.7239	0.0132	−0.7212	0.0132	−0.7236	0.0132
s_distdev	0.0416	0.0075	0.0462	0.0075	0.0431	0.0075	0.0449	0.0075	0.0433	0.0075
s_distwater	0.1220	0.0100	0.1303	0.0100	0.1372	0.0100	0.1330	0.0100	0.1328	0.0100

* “s_” indicates variable was scaled and centered.

**Table A11:** Results from the sensitivity analysis for varying random available sample size during the calving biological season (June–August) at the third order for adult female elk in southwestern Virginia from data collected January 2019 through November 2022. The used sample size was held constant at the number of true locations collected from the elk and available locations increased iteratively by 10 from 10 points per used to 50 points per used.

	Number Random Available Points per Real									
Full Model	10 Points		20 Points		30 Points		40 Points		50 Points	
Term *	Estimate	Std. Error	Estimate	Std. Error	Estimate	Std. Error	Estimate	Std. Error	Estimate	Std. Error
(Intercept)	−9.5115	0.0603	−10.2384	0.0610	−10.6522	0.0605	−10.9488	0.0609	−11.1730	0.0602
s_TPI	0.1974	0.0072	0.1959	0.0072	0.2008	0.0072	0.1991	0.0072	0.2003	0.0072
s_rough	−0.2759	0.0075	−0.2757	0.0075	−0.2794	0.0075	−0.2751	0.0075	−0.2783	0.0075
I(s_elevation^2^)	−0.0239	0.0034	−0.0226	0.0033	−0.0219	0.0033	−0.0229	0.0033	−0.0220	0.0033
s_distoak	0.1318	0.0070	0.1363	0.0068	0.1368	0.0068	0.1326	0.0068	0.1320	0.0068
s_distmh	0.2261	0.0076	0.2225	0.0075	0.2178	0.0075	0.2209	0.0075	0.2197	0.0075
s_distcon	−0.0777	0.0072	−0.0814	0.0072	−0.0809	0.0072	−0.0810	0.0072	−0.0798	0.0072
s_distot	0.0665	0.0080	0.0644	0.0079	0.0667	0.0080	0.0680	0.0080	0.0667	0.0080
s_distmine	−0.7814	0.0130	−0.7795	0.0130	−0.7810	0.0130	−0.7912	0.0131	−0.7855	0.0131
s_distdev	0.2407	0.0068	0.2438	0.0068	0.2432	0.0068	0.2417	0.0068	0.2419	0.0068
s_distwater	0.2651	0.0099	0.2610	0.0100	0.2618	0.0100	0.2640	0.0100	0.2667	0.0100

* “s_” indicates variable was scaled and centered.

**Table A12:** Results from the sensitivity analysis for varying random available sample size during the breeding biological season (September–November) at the third order for adult female elk in southwestern Virginia from data collected January 2019 through November 2022. The used sample size was held constant at the number of true locations collected from the elk and available locations increased iteratively by 10 from 10 points per used to 50 points per used.

	Number Random Available Points per Real									
Full Model	10 Points		20 Points		30 Points		40 Points		50 Points	
Term *	Estimate	Std. Error	Estimate	Std. Error	Estimate	Std. Error	Estimate	Std. Error	Estimate	Std. Error
(Intercept)	−9.5235	0.0711	−10.2462	0.0716	−10.6580	0.0715	−10.9519	0.0708	−11.1774	0.0707
s_TPI	0.2195	0.0073	0.2216	0.0073	0.2180	0.0073	0.2215	0.0073	0.2208	0.0073
s_rough	−0.3652	0.0077	−0.3629	0.0077	−0.3619	0.0077	−0.3607	0.0077	−0.3639	0.0077
I(s_elevation^2^)	0.0193	0.0032	0.0213	0.0031	0.0206	0.0031	0.0202	0.0031	0.0208	0.0031
s_distoak	0.1150	0.0073	0.1128	0.0073	0.1111	0.0072	0.1144	0.0072	0.1121	0.0072
s_distmh	0.2038	0.0082	0.2051	0.0082	0.2061	0.0081	0.2043	0.0082	0.2052	0.0081
s_distcon	−0.1818	0.0074	−0.1807	0.0075	−0.1791	0.0075	−0.1842	0.0075	−0.1775	0.0075
s_distot	0.0512	0.0066	0.0491	0.0067	0.0504	0.0067	0.0505	0.0067	0.0518	0.0067
s_distmine	−0.5879	0.0110	−0.5862	0.0111	−0.5825	0.0110	−0.5838	0.0110	−0.5833	0.0110
s_distdev	0.1775	0.0068	0.1759	0.0069	0.1769	0.0069	0.1754	0.0069	0.1740	0.0069
s_distwater	0.2743	0.0099	0.2752	0.0099	0.2675	0.0099	0.2729	0.0099	0.2740	0.0099

* “s_” indicates variable was scaled and centered.

**Table A13:** AICc table comparing models from the mid-gestation biological season (December–February) at the second order. Data for the models came from adult female elk in southwestern Virginia from January 2019 through November 2022. Presented are the model names, degrees of freedom (DF), log likelihood (logLik), Akaike Information Criterion with correction for small sample sizes (AICc), and the difference in AICc from the top model (Delta AICc).

Model	DF	logLik	AICc	Delta AICc
mg_full	12	−1,109,138	2,218,300.1235	0.0000
mg_m9	11	−1,109,156	2,218,333.1518	33.0283
mg_m8	10	−1,109,320	2,218,659.5331	359.4096
mg_m7	9	−1,109,554	2,219,126.2843	826.1608
mg_m6	8	−1,109,903	2,219,822.9986	1522.8751
mg_m5	7	−1,110,490	2,220,994.2325	2694.1090
mg_m4	6	−1,111,274	2,222,560.2445	4260.1210
mg_m3	5	−1,112,087	2,224,184.3847	5884.2612
mg_m2	4	−1,113,557	2,227,121.6298	8821.5063
mg_m1	3	−1,117,855	2,235,716.7532	17,416.6297
mg_null	2	−1,128,514	2,257,031.0982	38,730.9747

**Table A14:** Model definitions for models from the mid-gestation biological season (December–February) at the second order. Models referenced in Table A13.

Model	Definition *
mg_full	~s_distmine + s_distcon + s_distmh + s_distoak + s_distwater + I(s_elevation^2^) + s_rough + s_TPI + s_distot + s_distdev + (1|AID)
mg_m9	~s_distmine + s_distcon + s_distmh + s_distoak + s_distwater + I(s_elevation^2^) + s_rough + s_TPI + s_distot + (1|AID)
mg_m8	~s_distmine + s_distcon + s_distmh + s_distoak + s_distwater + I(s_elevation^2^) + s_rough + s_TPI + (1|AID)
mg_m7	~s_distmine + s_distcon + s_distmh + s_distoak + s_distwater + I(s_elevation^2^) + s_rough + (1|AID)
mg_m6	~s_distmine + s_distcon + s_distmh + s_distoak + s_distwater + I(s_elevation^2^) + (1|AID)
mg_m5	~s_distmine + s_distcon + s_distmh + s_distoak + s_distwater + (1|AID)
mg_m4	~s_distmine + s_distcon + s_distmh + s_distoak + (1|AID)
mg_m3	~s_distmine + s_distcon + s_distmh + (1|AID)
mg_m2	~s_distmine + s_distcon + (1|AID)
mg_m1	~s_distmine + (1|AID)
mg_null	~1 + (1|AID)

* “s_” indicates the variable was scaled and centered, “dist” indicates a distance variable, “mh” represents mixed hardwood forests, “mine” represents mines, “rough” represents terrain roughness, “oak” represents oak forests, “TPI” represents topographic position index, “con” represents conifer forests, “dev” represents developed areas, “ot” represents unmined open land, and AID is individual elk identity.

**Table A15:** AICc table comparing models from the late gestation biological season (March–May) at the second order. Data for the models came from adult female elk in southwestern Virginia from January 2019 through November 2022. Presented are the model names, degrees of freedom (DF), log likelihood (logLik), Akaike Information Criterion with correction for small sample sizes (AICc), and the difference in AICc from the top model (Delta AICc).

Model	DF	logLik	AICc	Delta AICc
lg_full	12	−1,015,353	2,030,730.1077	0.0000
lg_m9	11	−1,015,361	2,030,743.4772	13.3694
lg_m8	10	−1,015,464	2,030,947.2941	217.1864
lg_m7	9	−1,015,662	2,031,341.5907	611.4829
lg_m6	8	−1,015,895	2,031,806.8408	1076.7330
lg_m5	7	−1,016,174	2,032,361.8161	1631.7084
lg_m4	6	−1,016,629	2,033,270.4831	2540.3753
lg_m3	5	−1,018,043	2,036,095.4231	5365.3154
lg_m2	4	−1,019,538	2,039,084.3060	8354.1983
lg_m1	3	−1,023,146	2,046,298.5528	15,568.4450
lg_null	2	−1,030,382	2,060,767.8724	30,037.7647

**Table A16:** Model definitions for models from the late gestation biological season (March–May) at the second order. Models referenced in Table A15.

Model	Definition *
lg_full	~s_distmine + s_distcon + s_distoak + s_distwater + s_distmh + s_distot + s_TPI + I(s_elevation^2^) + s_rough + s_distdev + (1|AID)
lg_m9	~s_distmine + s_distcon + s_distoak + s_distwater + s_distmh + s_distot + s_TPI + I(s_elevation^2^) + s_rough + (1|AID)
lg_m8	~s_distmine + s_distcon + s_distoak + s_distwater + s_distmh + s_distot + s_TPI + I(s_elevation^2^) + (1|AID)
lg_m7	~s_distmine + s_distcon + s_distoak + s_distwater + s_distmh + s_distot + s_TPI + (1|AID)
lg_m6	~s_distmine + s_distcon + s_distoak + s_distwater + s_distmh + s_distot + (1|AID)
lg_m5	~s_distmine + s_distcon + s_distoak + s_distwater + s_distmh + (1|AID)
lg_m4	~s_distmine + s_distcon + s_distoak + s_distwater + (1|AID)
lg_m3	~s_distmine + s_distcon + s_distoak + (1|AID)
lg_m2	~s_distmine + s_distcon + (1|AID)
lg_m1	~s_distmine
lg_null	~1

* “s_” indicates variable was scaled and centered, “dist” indicates a distance variable, “mh” represents mixed hardwood forests, “mine” represents mines, “rough” represents terrain roughness, “oak” represents oak forests, “TPI” represents topographic position index, “con” represents conifer forests, “dev” represents developed areas, “ot” represents unmined open land, and AID is individual elk identity.

**Table A17:** AICc table comparing models from the calving biological season (June–August) at the second order. Data for the models came from adult female elk in southwestern Virginia from January 2019 through November 2022. Presented are the model names, degrees of freedom (DF), log likelihood (logLik), Akaike Information Criterion with correction for small sample sizes (AICc), and the difference in AICc from the top model (Delta AICc).

Model	DF	logLik	AICc	Delta AICc
calf_full	12	−984,168	1,968,360.6594	0.0000
calf_m9	11	−984,175	1,968,372.3593	11.6998
calf_m8	10	−984,226	1,968,471.8063	111.1468
calf_m7	9	−984,313	1,968,643.2352	282.5757
calf_m6	8	−984,495	1,969,006.8967	646.2372
calf_m5	7	−984,793	1,969,600.7951	1240.1356
calf_m4	6	−985,148	1,970,307.8180	1947.1585
calf_m3	5	−986,100	1,972,210.6845	3850.0250
calf_m2	4	−987,801	1,975,609.9333	7249.2739
calf_m1	3	−990,634	1,981,273.1589	12,912.4995
calf_null	2	−997,671	1,995,346.7970	26,986.1376

**Table A18:** Model definitions for models from the calving biological season (March–May) at the second order. Models referenced in Table A17.

Model	Definition *
calf_full	~s_distmine + s_distcon + s_distwater + s_distoak + I(s_elevation^2^) + s_TPI + s_distmh + s_rough + s_distdev + s_distot + (1|AID)
calf_m9	~s_distmine + s_distcon + s_distwater + s_distoak + I(s_elevation^2^) + s_TPI + s_distmh + s_rough + s_distdev + (1|AID)
calf_m8	~s_distmine + s_distcon + s_distwater + s_distoak + I(s_elevation^2^) + s_TPI + s_distmh + s_rough + (1|AID)
calf_m7	~s_distmine + s_distcon + s_distwater + s_distoak + I(s_elevation^2^) + s_TPI + s_distmh + (1|AID)
calf_m6	~s_distmine + s_distcon + s_distwater + s_distoak + I(s_elevation^2^) + s_TPI + (1|AID)
calf_m5	~s_distmine + s_distcon + s_distwater + s_distoak + I(s_elevation^2^) + (1|AID)
calf_m4	~s_distmine + s_distcon + s_distwater + s_distoak + (1|AID)
calf_m3	~s_distmine + s_distcon + s_distwater + (1|AID)
calf_m2	~s_distmine + s_distcon + (1|AID)
calf_m1	~s_distmine + (1|AID)
calf_null	~1 + (1|AID)

* “s_” indicates the variable was scaled and centered, “dist” indicates a distance variable, “mh” represents mixed hardwood forests, “mine” represents mines, “rough” represents terrain roughness, “oak” represents oak forests, “TPI” represents topographic position index, “con” represents conifer forests, “dev” represents developed areas, “ot” represents unmined open land, and AID is individual elk identity.

**Table A19:** AICc table comparing models from the breeding biological season (September–November) at the second order. Data for the models came from adult female elk in southwestern Virginia from January 2019 through November 2022. Presented are the model names, degrees of freedom (DF), log likelihood (logLik), Akaike Information Criterion with correction for small sample sizes (AICc), and the difference in AICc from the top model (Delta AICc).

Model	df	logLik	AICc	Delta AICc
breeding_full	12	−919,120	1,838,264.0605	0.0000
breeding_m9	11	−919,142	1,838,306.9887	42.9282
breeding_m8	10	−919,168	1,838,356.7785	92.7180
breeding_m7	9	−919,196	1,838,410.7041	146.6436
breeding_m6	8	−919,247	1,838,510.2233	246.1628
breeding_m5	7	−919,466	1,838,945.5308	681.4703
breeding_m4	6	−919,821	1,839,653.6596	1389.5991
breeding_m3	5	−920,936	1,841,882.1869	3618.1264
breeding_m2	4	−922,336	1,844,680.9301	6416.8696
breeding_m1	3	−924,304	1,848,613.1510	10,349.0905
breeding_null	2	−932,250	1,864,504.6465	26,240.5860

**Table A20:** Model definitions for models from the breeding biological season (September–November) at the second order. Models referenced in Table A19.

Model	Definition *
breeding_full	~s_distmine + s_distcon + s_distwater + s_distoak + s_distmh + s_TPI + s_rough + s_distdev + s_distot + I(s_elevation^2^) + (1|AID)
breeding_m9	~s_distmine + s_distcon + s_distwater + s_distoak + s_distmh + s_TPI + s_rough + s_distdev + s_distot + (1|AID)
breeding_m8	~s_distmine + s_distcon + s_distwater + s_distoak + s_distmh + s_TPI + s_rough + s_distdev + (1|AID)
breeding_m7	~s_distmine + s_distcon + s_distwater + s_distoak + s_distmh + s_TPI + s_rough + (1|AID)
breeding_m6	~s_distmine + s_distcon + s_distwater + s_distoak + s_distmh + s_TPI + (1|AID)
breeding_m5	~s_distmine + s_distcon + s_distwater + s_distoak + s_distmh + (1|AID)
breeding_m4	~s_distmine + s_distcon + s_distwater + s_distoak + (1|AID)
breeding_m3	~s_distmine + s_distcon + s_distwater + (1|AID)
breeding_m2	~s_distmine + s_distcon + (1|AID)
breeding_m1	~s_distmine + (1|AID)
breeding_null	~1 + (1|AID)

* “s_” indicates the variable was scaled and centered, “dist” indicates a distance variable, “mh” represents mixed hardwood forests, “mine” represents mines, “rough” represents terrain roughness, “oak” represents oak forests, “TPI” represents topographic position index, “con” represents conifer forests, “dev” represents developed areas, “ot” represents unmined open land, and AID is individual elk identity.

**Table A21:** AICc table comparing models from the mid-gestation biological season (December–February) at the third order. Data for the models came from adult female elk in southwestern Virginia from January 2019 through November 2022. Presented are the model names, degrees of freedom (DF), log likelihood (logLik), Akaike Information Criterion with correction for small sample sizes (AICc), and the difference in AICc from the top model (Delta AICc).

Model	DF	logLik	AICc	Delta AICc
mg_full	12	−167,996	336,015.4354	0.0000
mg_m9	11	−168,004	336,030.8815	15.4461
mg_m8	10	−168,021	336,062.4390	47.0036
mg_m7	9	−168,074	336,166.9275	151.4921
mg_m6	8	−168,169	336,353.9112	338.4758
mg_m5	7	−168,343	336,699.8212	684.3858
mg_m4	6	−168,622	337,255.1907	1239.7554
mg_m3	5	−168,937	337,884.4442	1869.0089
mg_m2	4	−169,802	339,612.0065	3596.5711
mg_m1	3	−170,890	341,785.8925	5770.4571
mg_null	2	−174,663	349,330.7352	13,315.2998

**Table A22:** Model definitions for models from the mid-gestation biological season (December–February) at the second order. Models referenced in Table A21.

Model	Definition *
mg_full	~s_distmh + s_distmine + s_rough + s_distoak + I(s_elevation^2^) + s_TPI + s_distwater + s_distcon + s_distdev + s_distot + (1|AID)
mg_m9	~s_distmh + s_distmine + s_rough + s_distoak + I(s_elevation^2^) + s_TPI + s_distwater + s_distcon + s_distdev + (1|AID)
mg_m8	~s_distmh + s_distmine + s_rough + s_distoak + I(s_elevation^2^) + s_TPI + s_distwater + s_distcon + (1|AID)
mg_m7	~s_distmh + s_distmine + s_rough + s_distoak + I(s_elevation^2^) + s_TPI + s_distwater + (1|AID)
mg_m6	~s_distmh + s_distmine + s_rough + s_distoak + I(s_elevation^2^) + s_TPI + (1|AID)
mg_m5	~s_distmh + s_distmine + s_rough + s_distoak + I(s_elevation^2^) + (1|AID)
mg_m4	~s_distmh + s_distmine + s_rough + s_distoak + (1|AID)
mg_m3	~s_distmh + s_distmine + s_rough + (1|AID)
mg_m2	~s_distmh + s_distmine + (1|AID)
mg_m1	~s_distmh + (1|AID)
mg_null	~1 + (1|AID)

* “s_” indicates the variable was scaled and centered, “dist” indicates a distance variable, “mh” represents mixed hardwood forests, “mine” represents mines, “rough” represents terrain roughness, “oak” represents oak forests, “TPI” represents topographic position index, “con” represents conifer forests, “dev” represents developed areas, “ot” represents unmined open land, and AID is individual elk identity.

**Table A23:** AICc table comparing models from the late gestation biological season (March–June) at the third order. Data for the models came from adult female elk in southwestern Virginia from January 2019 through November 2022. Presented are the model names, degrees of freedom (DF), log likelihood (logLik), Akaike Information Criterion with correction for small sample sizes (AICc), and the difference in AICc from the top model (Delta AICc).

Model	DF	logLik	AICc	Delta AICc
lg_full	12	−237,903	475,830.5926	0.0000
lg_m9	11	−237,918	475,858.6988	28.1062
lg_m8	10	−237,932	475,884.2451	53.6525
lg_m7	9	−238,014	476,046.0037	215.4111
lg_m6	8	−238,090	476,196.9555	366.3629
lg_m5	7	−238,264	476,542.4428	711.8503
lg_m4	6	−238,458	476,927.6107	1097.0181
lg_m3	5	−238,704	477,418.7256	1588.1331
lg_m2	4	−239,723	479,454.0233	3623.4308
lg_m1	3	−242,486	484,978.1387	9147.5461
lg_null	2	−247,488	494,979.8558	19,149.2633

**Table A24:** Model definitions for models from the late gestation biological season (March–May) at the second order. Models referenced in Table A23.

Model	Definition *
lg_full	~s_distmh + s_distmine + s_rough + s_TPI + s_distoak + I(s_elevation^2^) + s_distcon + s_distwater + s_distdev + s_distot + (1|AID)
lg_m9	~s_distmh + s_distmine + s_rough + s_TPI + s_distoak + I(s_elevation^2^) + s_distcon + s_distwater + s_distdev + (1|AID)
lg_m8	~s_distmh + s_distmine + s_rough + s_TPI + s_distoak + I(s_elevation^2^) + s_distcon + s_distwater + (1|AID)
lg_m7	~s_distmh + s_distmine + s_rough + s_TPI + s_distoak + I(s_elevation^2^) + s_distcon + (1|AID)
lg_m6	~s_distmh + s_distmine + s_rough + s_TPI + s_distoak + I(s_elevation^2^) + (1|AID)
lg_m5	~s_distmh + s_distmine + s_rough + s_TPI + s_distoak + (1|AID)
lg_m4	~s_distmh + s_distmine + s_rough + s_TPI + (1|AID)
lg_m3	~s_distmh + s_distmine + s_rough + (1|AID)
lg_m2	~s_distmh + s_distmine + (1|AID)
lg_m1	~s_distmh + (1|AID)
lg_null	~1 + (1|AID)

* “s_” indicates the variable was scaled and centered, “dist” indicates a distance variable, “mh” represents mixed hardwood forests, “mine” represents mines, “rough” represents terrain roughness, “oak” represents oak forests, “TPI” represents topographic position index, “con” represents conifer forests, “dev” represents developed areas, “ot” represents unmined open land, and AID is individual elk identity.

**Table A25:** AICc table comparing models from the calving biological season (March–June) at the third order. Data for the models came from adult female elk in southwestern Virginia from January 2019 through November 2022. Presented are the model names, degrees of freedom (DF), log likelihood (logLik), Akaike Information Criterion with correction for small sample sizes (AICc), and the difference in AICc from the top model (Delta AICc).

Model	DF	logLik	AICc	Delta AICc
calf_full	12	−234,507	469,037.8703	0.0000
calf_m9	11	−234,531	469,084.8237	46.9534
calf_m8	10	−234,561	469,141.5025	103.6322
calf_m7	9	−234,621	469,259.0082	221.1379
calf_m6	8	−234,801	469,618.9847	581.1144
calf_m5	7	−235,096	470,206.3390	1168.4686
calf_m4	6	−235,397	470,806.6329	1768.7626
calf_m3	5	−236,095	472,200.1925	3162.3222
calf_m2	4	−236,746	473,499.4662	4461.5958
calf_m1	3	−238,577	477,159.7001	8121.8298
calf_null	2	−242,298	484,599.7119	15,561.8416

**Table A26:** Model definitions for models from the calving biological season (June–August) at the second order. Models referenced in Table A25.

Model	Definition *
calf_full	~s_distmine + s_distmh + s_rough + s_distdev + s_distwater + s_TPI + s_distoak + s_distcon + s_distot + I(s_elevation^2^) + (1|AID)
calf_m9	~s_distmine + s_distmh + s_rough + s_distdev + s_distwater + s_TPI + s_distoak + s_distcon + s_distot + (1|AID)
calf_m8	~s_distmine + s_distmh + s_rough + s_distdev + s_distwater + s_TPI + s_distoak + s_distcon + (1|AID)
calf_m7	~s_distmine + s_distmh + s_rough + s_distdev + s_distwater + s_TPI + s_distoak + (1|AID)
calf_m6	~s_distmine + s_distmh + s_rough + s_distdev + s_distwater + s_TPI + (1|AID)
calf_m5	~s_distmine + s_distmh + s_rough + s_distdev + s_distwater + (1|AID)
calf_m4	~s_distmine + s_distmh + s_rough + s_distdev + (1|AID)
calf_m3	~s_distmine + s_distmh + s_rough + (1|AID)
calf_m2	~s_distmine + s_distmh + (1|AID)
calf_m1	~s_distmine + (1|AID)
calf_null	~1 + (1|AID)

* “s_” indicates the variable was scaled and centered, “dist” indicates a distance variable, “mh” represents mixed hardwood forests, “mine” represents mines, “rough” represents terrain roughness, “oak” represents oak forests, “TPI” represents topographic position index, “con” represents conifer forests, “dev” represents developed areas, “ot” represents unmined open land, and AID is individual elk identity.

**Table A27:** AICc table comparing models from the breeding biological season (September–November) at the third order. Data for the models came from adult female elk in southwestern Virginia from January 2019 through November 2022. Presented are the model names, degrees of freedom (DF), log likelihood (logLik), Akaike Information Criterion with correction for small sample sizes (AICc), and the difference in AICc from the top model (Delta AICc).

Model	DF	logLik	AICc	Delta AICc
breeding_full	12	−232,886	465,795.7808	0
breeding_m9	11	−232,908	465,838.0732	42.29237
breeding_m8	10	−232,936	465,891.0841	95.30324
breeding_m7	9	−233,054	466,126.1389	330.3581
breeding_m6	8	−233,328	466,671.4326	875.6518
breeding_m5	7	−233,672	467,358.4079	1562.627
breeding_m4	6	−234,127	468,265.5934	2469.813
breeding_m3	5	−234,580	469,170.2200	3374.439
breeding_m2	4	−235,497	471,001.8457	5206.065
breeding_m1	3	−237,253	474,511.8185	8716.038
breeding_null	2	−239,954	479,911.2015	14,115.42

**Table A28:** Model definitions for models from the breeding biological season (September–November) at the third order. Models referenced in Table A27.

Model	Definition *
breeding_full	~s_distmine + s_rough + s_distmh + s_distwater + s_TPI + s_distdev + s_distcon + s_distoak + s_distot + I(s_elevation^2^) + (1|AID)
breeding_m9	~s_distmine + s_rough + s_distmh + s_distwater + s_TPI + s_distdev + s_distcon + s_distoak + s_distot + (1|AID)
breeding_m8	~s_distmine + s_rough + s_distmh + s_distwater + s_TPI + s_distdev + s_distcon + s_distoak + (1|AID)
breeding_m7	~s_distmine + s_rough + s_distmh + s_distwater + s_TPI + s_distdev + s_distcon + (1|AID)
breeding_m6	~s_distmine + s_rough + s_distmh + s_distwater + s_TPI + s_distdev + (1|AID)
breeding_m5	~s_distmine + s_rough + s_distmh + s_distwater + s_TPI + (1|AID)
breeding_m4	~s_distmine + s_rough + s_distmh + s_distwater + (1|AID)
breeding_m3	~s_distmine + s_rough + s_distmh + (1|AID)
breeding_m2	~s_distmine + s_rough + (1|AID)
breeding_m1	~s_distmine + (1|AID)
breeding_null	~1 + (1|AID)

* “s_” indicates the variable was scaled and centered, “dist” indicates a distance variable, “mh” represents mixed hardwood forests, “mine” represents mines, “rough” represents terrain roughness, “oak” represents oak forests, “TPI” represents topographic position index, “con” represents conifer forests, “dev” represents developed areas, “ot” represents unmined open land, and AID is individual elk identity.

**Table A29:** Top generalized linear mixed-effects models examining resource selection at the second order for female elk in southwestern Virginia, USA from March 2019– November 2022 based on corrected delta AIC. Land cover types were implemented via a distance-based approach. Variables are listed in order of explanatory power.

Season	Term *	Estimate	Std. Error	z Value	Pr(>|z|)
Late Gestation	Mines_1_	−0.411	0.005	−77.935	<0.001
	Conifer_1_	−0.304	0.004	−79.444	<0.001
	Oak_1_	0.170	0.003	53.947	<0.001
	Water_1_	0.158	0.003	47.035	<0.001
	Mixed Hardwood_1_	0.083	0.004	22.953	<0.001
	Unmined Open Land_1_	−0.090	0.004	−20.218	<0.001
	TPI_2_	0.078	0.003	22.504	<0.001
	Elevation^2^_2_	−0.035	0.002	−17.685	<0.001
	Roughness_2_	−0.051	0.003	−14.534	<0.001
	Developed_1_	−0.014	0.004	−3.911	<0.001
Calving	Mines_1_	−0.473	0.005	−87.575	<0.001
	Conifer_1_	−0.266	0.004	−69.089	<0.001
	Water_1_	0.189	0.003	55.638	<0.001
	Oak_1_	0.143	0.003	43.768	<0.001
	Elevation^2^_2_	−0.049	0.002	−23.281	<0.001
	TPI_2_	0.074	0.004	20.975	<0.001
	Mixed Hardwood_1_	0.062	0.004	16.543	<0.001
	Roughness_2_	−0.047	0.004	−13.230	<0.001
	Developed_1_	−0.032	0.004	−8.810	<0.001
	Unmined Open Land_1_	−0.016	0.004	−3.685	<0.001
Breeding	Mines_1_	−0.526	0.006	−91.685	<0.001
	Conifer_1_	−0.231	0.004	−58.385	<0.001
	Water_1_	0.187	0.004	52.508	<0.001
	Oak_1_	0.153	0.003	45.864	<0.001
	Mixed Hardwood_1_	0.070	0.004	18.273	<0.001
	TPI_2_	0.077	0.004	21.281	<0.001
	Roughness_2_	−0.031	0.004	−8.367	<0.001
	Developed_1_	0.034	0.004	9.267	<0.001
	Unmined Open Land_1_	−0.031	0.004	−6.981	<0.001
	Elevation^2^_2_	−0.012	0.002	−6.595	<0.001
Mid−gestation	Mines_1_	−0.510	0.005	−96.987	<0.001
	Conifer_1_	−0.301	0.004	−82.767	<0.001
	Mixed Hardwood_1_	0.100	0.003	29.705	<0.001
	Oak_1_	0.154	0.003	51.168	<0.001
	Water_1_	0.116	0.003	35.219	<0.001
	Elevation^2^_2_	−0.059	0.002	−28.519	<0.001
	Roughness_2_	−0.087	0.003	−25.971	<0.001
	TPI_2_	0.073	0.003	21.857	<0.001
	Unmined Open Land_1_	−0.077	0.004	−18.606	<0.001
	Developed_1_	0.020	0.003	5.941	<0.001

* Subscript 1 indicates a distance variable, subscript 2 indicates a point condition variable.

**Table A30:** Top generalized linear mixed-effects models examining resource selection at the third order for female elk in southwestern Virginia, USA from March 2019–November 2022 based on corrected delta AIC. Land cover types were implemented via a distance-based approach. Variables are listed in order of explanatory power.

Season	Term *	Estimate	Std. Error	z Value	Pr(>|z|)
Late Gestation	Mixed Hardwood_1_	0.339	0.008	44.208	<0.001
	Mines_1_	−0.720	0.013	−54.607	<0.001
	Roughness_2_	−0.293	0.008	−38.372	<0.001
	TPI_2_	0.183	0.007	25.015	<0.001
	Oak_1_	0.158	0.007	23.315	<0.001
	Elevation^2^_2_	−0.075	0.005	−16.438	<0.001
	Conifer_1_	−0.093	0.007	−12.769	<0.001
	Water_1_	0.130	0.010	13.056	<0.001
	Developed_1_	0.046	0.007	6.158	<0.001
	Unmined open land_1_	−0.042	0.008	−5.312	<0.001
Calving	Mines_1_	−0.779	0.013	−60.183	<0.001
	Mixed Hardwood_1_	0.223	0.007	29.683	<0.001
	Roughness_2_	−0.276	0.008	−36.673	<0.001
	Developed_1_	0.244	0.007	35.720	<0.001
	Water_1_	0.261	0.010	26.144	<0.001
	TPI_2_	0.196	0.007	27.194	<0.001
	Oak_1_	0.136	0.007	19.933	<0.001
	Conifer_1_	−0.081	0.007	−11.244	<0.001
	Unmined open land_1_	0.064	0.008	8.123	<0.001
	Elevation^2^_2_	−0.023	0.003	−6.786	<0.001
Breeding	Mines_1_	−0.586	0.011	−53.031	<0.001
	Roughness_2_	−0.363	0.008	−47.103	<0.001
	Mixed Hardwood_1_	0.205	0.008	24.969	<0.001
	Water_1_	0.275	0.010	27.832	<0.001
	TPI_2_	0.222	0.007	30.467	<0.001
	Developed_1_	0.176	0.007	25.534	<0.001
	Conifer_1_	−0.181	0.007	−24.250	<0.001
	Oak_1_	0.113	0.007	15.398	<0.001
	Unmined open land_1_	0.049	0.007	7.325	<0.001
	Elevation^2^_2_	0.021	0.003	6.781	<0.001
Mid-gestation	Mixed Hardwood_1_	0.340	0.009	38.164	<0.001
	Mines_1_	−0.397	0.012	−31.843	<0.001
	Roughness_2_	−0.315	0.009	−34.261	<0.001
	Oak_1_	0.249	0.008	32.356	<0.001
	Elevation^2^_2_	−0.108	0.006	−17.410	<0.001
	TPI_2_	0.162	0.009	18.518	<0.001
	Water_1_	0.174	0.012	14.922	<0.001
	Conifer_1_	−0.090	0.009	−10.331	<0.001
	Developed_1_	0.059	0.009	6.631	<0.001
	Unmined open land_1_	−0.040	0.010	−4.099	<0.001

* Subscript 1 indicates a distance variable, subscript 2 indicates a point condition variable.

**Table A31:** Second order k-fold cross validation results for the top models from each of the biological seasons. Data for the models came from adult female elk in southwestern Virginia from January 2019 through November 2022. For this validation, we used k = 4, 100 repetitions, and 10 bins.

Season	Observed Mean	Observed Std. Dev.	Random Mean	Random Std. Dev.
Late Gestation	1.0000	0.0000	−0.0293	0.3209
Calving	1.0000	0.0000	0.0046	0.3203
Breeding	0.9999	0.0012	−0.0051	0.3508
Mid-gestation	1.0000	0.0000	−0.0268	0.3748

**Table A32:** Third order k-fold cross validation results for the top models from each of the biological seasons. Data for the models came from adult female elk in southwestern Virginia from January 2019 through November 2022. For this validation, we used k = 4, 100 repetitions, and 10 bins.

Season	Observed Mean	Observed Std. Dev.	Random Mean	Random Std. Dev.
Late Gestation	0.9328	0.0258	−0.0036	0.3276
Calving	0.9842	0.0111	0.0141	0.3472
Breeding	0.9767	0.0189	0.0333	0.3561
Mid-gestation	0.9400	0.0233	−0.0112	0.3045
